# Computer-Based Experiment for the Motion of Spring Oscillator on a Linear Air Track Using Ultrasonic Sensor

**DOI:** 10.3390/s24144441

**Published:** 2024-07-09

**Authors:** Bin Wu, Yiqing Xu, Guoquan Zhou, Yan Fan

**Affiliations:** 1 College of Mathematics and Computer Science, Zhejiang A&F University, Hangzhou 311300, China; 2 College of Optical, Mechanical and Electrical Engineering, Zhejiang A&F University, Hangzhou 311300, China; xuyiqing136@zafu.edu.cn (Y.X.); 19920006@zafu.edu.cn (G.Z.); 20060068@zafu.edu.cn (Y.F.)

**Keywords:** ultrasonic sensor, Arduino, LabVIEW, simple harmonics motion, distance measurement

## Abstract

In the present paper, an affordable innovative physical experimental equipment consisting of an upper computer, an ultrasonic sensor module, and an Arduino microcontroller has been designed. The relationship between the position of the slider fixed on two springs and time is measured by using the ultrasonic sensor module. A system for slider motion data and image acquisition is constructed by using the LabVIEW interface of Arduino UNO R3. The purpose of this experiment is to demonstrate and interpret the propagation of waves represented by harmonic motion. The spring oscillator system including a slider and two springs is measured and recorded, and the motion can be realized using curve fitting to the wave equation in Sigmaplot. The vibration periods obtained from experimental measurements and curve fitting of the wave equation are 1.130 s and 1.165 s, respectively. The experimental data are in good agreement with the theoretical model. The experimental measurement results show that the maximum kinetic energy is 0.0792 J, the maximum potential energy is 0.0795 J, and the total energy at the position of half the amplitude is 0.0791 J. The results verify the mechanical energy conservation of spring oscillator system in a short time. This self-made instrument has improved the visualization and the automation level of the corresponding experiments.

## 1. Introduction

The wave of digitization and artificial intelligence has swept across the world, with big data and widespread applications such as ChatGPT and the metaverse everywhere. The digitization and informatization of experimental teaching has become a hot topic in the current global teaching development. Modern technology plays a crucial role in the digital world and is also used to help improve academic performance in scientific knowledge [1,2]. Physical instruments require a computer interface system that integrates data collection, processing, regulation, and presentation [3]. Arduino is an excellent microcontroller that can be used for much more than just creating interactive projects. It is a low-cost open-source electronic platform based on easy-to-use hardware and software. It has been utilized in countless of projects, from simple objects to complex scientific instruments [4,5,6,7,8]. LabVIEW 2018 is a system engineering software designed for applications that requires quick access to hardware and data insights for testing, measurement, and control. Its name stands for “laboratory virtual instrument engineering workbench” and is widely used in industrial manufactory, engineering, and physics. By combining LabVIEW graphical programming language with the Arduino open-source hardware ecosystem, a cost-effective prototype of a physical experimental device has been developed.

Ultrasound is defined as sound waves with a frequency greater than 20 kHz. The frequency range of ultrasound covers tens of kHz to tens of MHz. This form of mechanical wave possesses notable attributes such as high penetrating power, precise directionality, and efficient concentration of sound energy [9,10,11,12,13]. Because of its benefits such as contactless measurement, affordability, convenient operation, and fast measurement, it has been successfully applied in many scenarios, such as ultrasonic detection, safety alarms, and robot obstacle avoidance [14,15,16,17,18,19,20]. Researchers are particularly interested in ultrasonic ranging technology because using ultrasonic technology for distance measurement can significantly improve measurement accuracy and stability. This progress makes ultrasonic ranging more appropriate for industrial control systems and high-level instruments [21,22,23,24,25,26,27,28,29]. In this paper, ultrasonic sensors are used to measure the distance of the slider on a linear air track.

The motion of spring oscillator on a linear air track experiment is a common mechanical experiment offered in college physics. The air track is mainly used for mechanical experiments and can be used for related research on object motion, velocity, acceleration, and linear momentum. The original air track is equipped with a digital timer with two photoelectric gates and corresponding accessories for all experimental data measurements. The traditional method of this experiment can only measure the period and energy of harmonic vibration through a timing counter, and cannot achieve visualization of the results. In order to enhance students’ comprehension of physics theory, the utilization of visualized data obtained from experiments can facilitate conceptualization and promote a deeper understanding of the subject matter. To achieve this goal, an innovative, inexpensive, up-to-date, and easy-to-use physical experimental setup is designed. This designed device can conduct experimental research on harmonic motion, and the collected images can also be compared and analyzed with theoretical wave equations. When used for the study of harmonic motion, the position of the slider fixed on two springs is represented as a function of time. The real-time data obtained from ultrasonic sensors for measuring harmonic motion are synchronously displayed on the front panel of LabVIEW. Then, the information needs to be analyzed to extract valuable insights. Sigmaplot 15 is used for the visualization and curve fitting of the collected data, and the mechanical energy conservation of spring oscillatory system in a short period of time is verified. The completed experiments indicate that the designed equipment makes the process of measurement intuitive and accurate, and it can better meet the requirements of scientific research test and teaching experiment.

The major contributions of this article are stated as follows:

(1) The self-made physics lab apparatus based on LabVIEW interface for Arduino has significantly improved the visualization and automation of the experiment. The physical devices can not only perform point measurement, but also support continuous measurement, achieving the real-time measurement of dynamic amplitude, data visualization, and a more convenient observation of experimental details and quantitative calculation.
(2) Through this experimental platform, students have practiced the STEM education philosophy, which integrates disciplines from four fields: science (physics concept), technology (electronic circuit design and computer programming), engineering (design and build apparatus), and mathematics (investigate data using Sigmaplot). The aim is to cultivate students’ innovative thinking, problem-solving, and teamwork abilities.

## 2. Experimental Principles

### 2.1. Harmonic Vibration Equation

Simple harmonic vibration is the most basic and simplest form of vibration, and all complex vibrations can be decomposed into several simple harmonic vibrations. Therefore, simple harmonic vibration is the basis for studying other complex vibrations. The period and energy of harmonic vibration are the measured quantities in this experiment.

**Figure 1:**
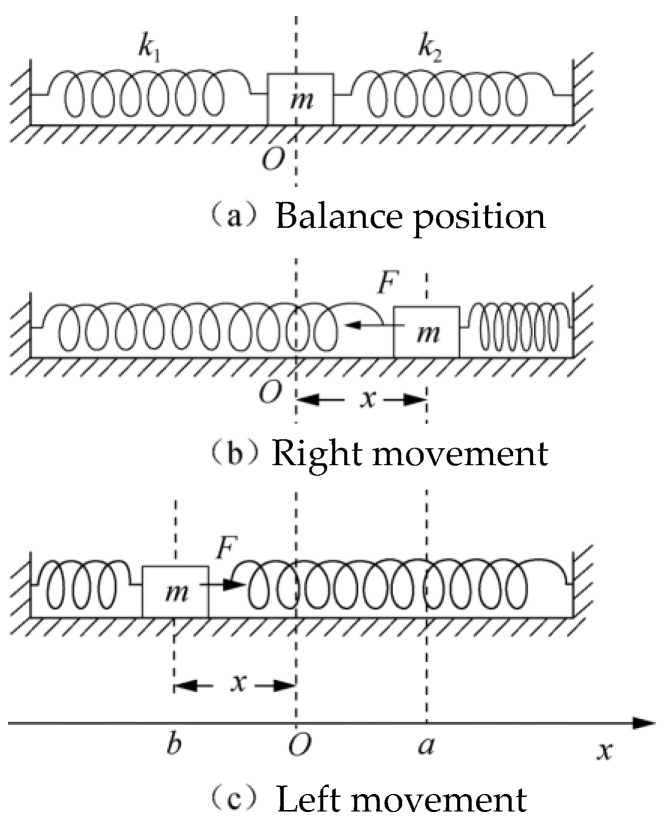
Schematic diagram of spring oscillator.

A slider with a mass of *m* is placed on an air track while it is level and ventilated. Two springs with stiffness coefficients of *k*_1_ and *k*_2_ are connected to each end of the slider, and the other end of the two springs is fixed at both ends of the air rail, as shown in Figure 1a. This device is called a spring oscillator. At the beginning, the slider is stationary at the equilibrium position “*O*” point, and at this point, the combined force on the slider *F* = 0. If the slider is pulled by hand to deviate from the equilibrium position, as shown in Figure 1b, and then released, the slider will undergo periodic reciprocating motion near the equilibrium position. When the displacement of the slider relative to the equilibrium point is *x*, the resulting force is as follows:

(1) $$F=-\left(k_{1}+k_{2}\right)x$$

According to Newton’s second law, it can be concluded that

(2) $$m\frac{d^{2}x}{dt^{2}}=-\left(k_{1}+k_{2}\right)x$$

Then, a new parameter *ω* is introduced and defined as follows:

(3) $$\omega =\sqrt{\frac{k_{1}+k_{2}}{m}}$$

where the physical meaning of *ω* is angular frequency. Accordingly, Equation (2) becomes the following form:

(4) $$\frac{d^{2}x}{dt^{2}}=-\omega^{2}x$$

The solution of this differential equation, *x*(*t*), whose second derivative is proportional to the negative of the function itself, will be a function that oscillates back and forth with time. Therefore, the solution to Equation (4) is expressed in the analytical form as

(5) $$x=A\cos\left(\omega t+\varphi_{0}\right)$$

Equation (5) indicates that the slider undergoes harmonic vibration. In Equation (5), *A* is the amplitude and $\varphi_{0}$ is the initial phase.

The vibration period of the system is defined as

(6) $$T=\frac{2\pi }{\omega }=2\pi \sqrt{\frac{m}{k_{1}+k_{2}}}$$

In the previous experiment, the slider vibrates harmoniously on a linear air track. A single light blocking plate is used to block the light twice, and the digital timer records the time of vibration once, which is the period *T*.

If the displacement *x* and velocity at a certain moment can be measured, then the kinetic energy and potential energy of the spring oscillator are calculated by $E_{k}^{\prime }=mv^{2}/2$ and $E_{p}^{\prime }=kx^{2}/2$, respectively. At this point, the mechanical energy of the spring oscillator is

(7) $$E=E_{k}^{\prime }+E_{p}^{\prime }=mv^{2}/2+kx^{2}/2$$

This is due to $x=A\cos\left(\omega t+\varphi_{0}\right)$ and $v=A\omega \sin\left(\omega t+\varphi_{0}\right)$. By substituting the above equation, one can obtain

(8) $$E=mA^{2}\omega^{2}/2=kA^{2}/2$$

Similarly, it can be concluded that the kinetic energy is maximized when the slider moves to the equilibrium position “*O*”, which is listed as follows:

(9) $$E_{k}=mv_{\max}^{2}/2$$

The potential energy of the slider reaches the maximum value at its maximum displacement (or amplitude), which is found to be

(10) $$E_{p}=\frac{1}{2}\left(k_{1}+k_{2}\right)A^{2}$$

According to the law of conservation of mechanical energy, the following conclusion can be drawn:

(11) $$E=E_{k}=E_{p}$$

Therefore, this experiment can also verify the law of conservation of mechanical energy.

### 2.2. Principles of Ultrasonic Distance Measurement

**Figure 2:**
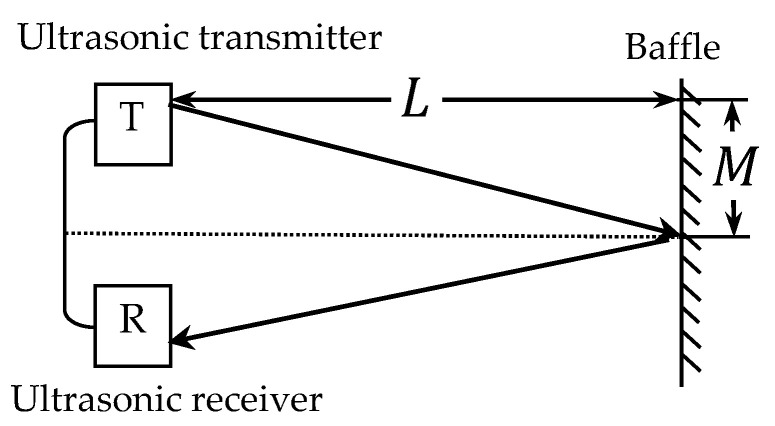
Schematic diagram of ultrasonic distance measurement.

Due to the frequency of ultrasound being greater than 20 kHz, it has concentrated sound energy, good directional propagation, and is not affected by electromagnetic interference. Therefore, ultrasonic distance detection is an effective non-contact distance measurement method. There are various methods for ultrasonic ranging, and in this experiment, the pulse echo method is used. The principle of distance measurement is shown in Figure 2. The transmitter sends a beam of ultrasonic waves, and at the same time, the timer in the microcontroller starts counting. The signal reflected by obstacles in the ultrasonic wave is received by the receiver, and the timing of the microcontroller timer is stopped when the echo is received. The speed of ultrasonic wave propagation in air is approximately 340 m/s, which is equivalent to the speed of sound. To be precise, the ultrasound speed is determined by the medium, and the speed in the air is calculated using the following formula:

(12) $$c=331.45\sqrt{1+T/273.15}$$

where *c* is the ultrasonic wave propagation speed in the air and *T* represents room temperature. The time taken from the emission of ultrasonic waves to the receipt of echoes is recorded as *t*, which is obtained through the timer in the Arduino microcontroller. The distance *L* between the baffle and the transmitter can be calculated using the following formula:

(13) $$L=\sqrt{{\left(\frac{1}{2}ct\right)}^{2}-M^{2}}$$

## 3. System Design Scheme

### 3.1. General Scheme

**Figure 3:**
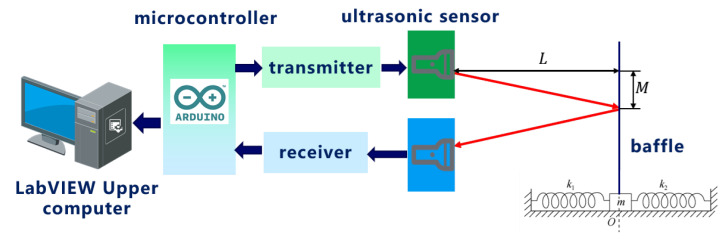
Overall system design scheme diagram.

The experimental setup mainly consists of four parts: upper computer (installed LabVIEW), Arduino microcontroller, HC-SR04 ultrasonic sensor, and spring oscillator with baffle suspended on a linear air track. The design scheme of the entire system is shown in Figure 3.

**Figure 4:**
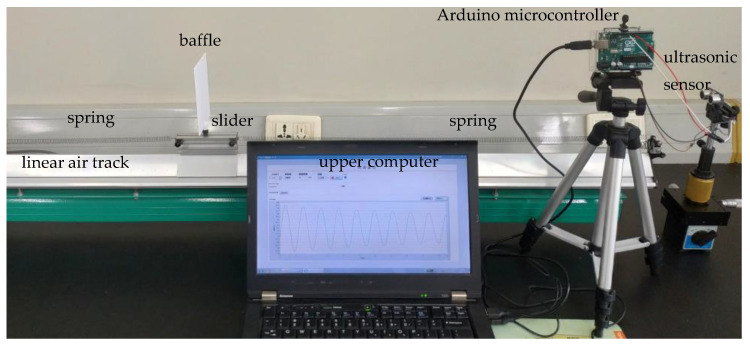
Photo of digital harmonic vibration device.

During the experiment, the baffle on the slider is installed with its center facing the ultrasonic sensor. The air pump is turned on to make the slider vibrate harmoniously, as shown in Figure 4. While the system is running, LabVIEW2018’s NI-VISA window configuration assistant gathers data from an ultrasonic sensor via Arduino. It then processes, analyzes, stores, displays, and conducts calculations on the data to visualize harmonic vibration. On this basis, quantitative analysis is conducted by establishing coordinate axes, benchmarking, and combining visual images and tables. By studying the motion images of spring oscillators over a short period of time, the conservation of mechanical energy in the spring oscillator system can be verified.

### 3.2. Hardware Design Scheme

#### 3.2.1. Master Controller

**Figure 5:**
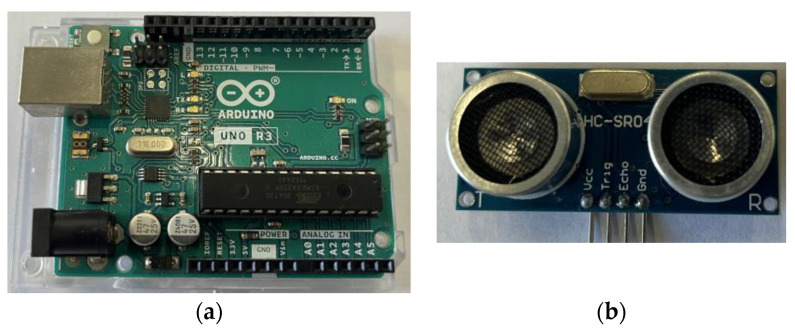
The hardware of the system. (**a**) Arduino UNO control board; (**b**) HC-SR04 ultrasonic sensor.

At present, the word “maker” is deeply rooted in people’s hearts, and the Arduino platform is the first choice of electronic makers. This system uses the most cost-effective UNO control board in Arduino, which has abundant pins, high efficiency of software program development, rich library functions, open-source hardware products, and low price. Its core processing unit is ATmega328P, which mainly includes 14 I/O interfaces, supports PWM, six analog input interfaces, and one serial communication interface. It can fully meet the design requirements of this experiment, as shown in Figure 5a.

#### 3.2.2. Sensor

The HC-SR04 ultrasonic sensor is used for distance measurement in this experiment, as shown in Figure 5b. It employs a dual-probe configuration renowned for its robust anti-interference capabilities. It is capable of delivering non-contact distance measurements ranging from 2 to 500 cm with an impressive accuracy of up to 3 mm. This particular model of ultrasonic sensor is composed primarily of three integral components: an ultrasonic transmitter, a receiver, and a control circuit. Upon the TRIG trigger pin emitting a high-level signal of at least 10 μs, the module automatically dispatches eight 40 kHz square waves and initiates an automatic detection for any returning signals. Once a signal is detected, it will manifest as a high-level output via the ECHO pin. The duration of this high-level pulse corresponds to the time taken for the ultrasonic wave to travel and return. The control circuitry then leverages the measured time difference to calculate the distance from the baffle to the sensor. 

### 3.3. Software Design Scheme

#### 3.3.1. Arduino Program Design

**Figure 6:**
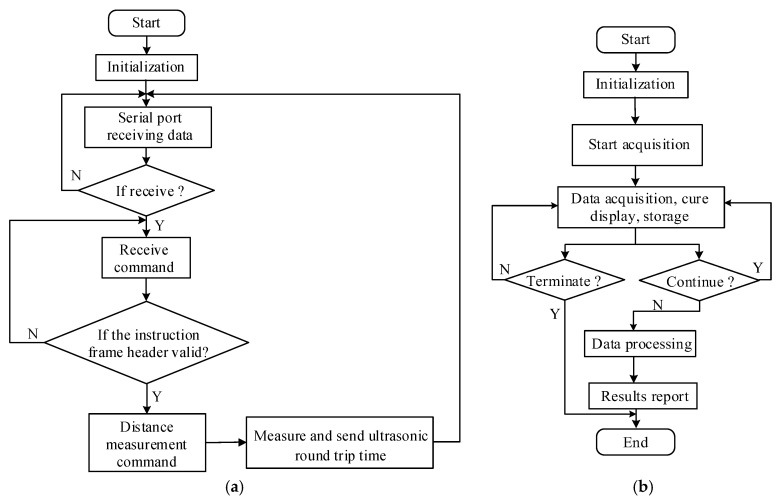
The flowchart of the software. (**a**) Flowchart of Arduino program; (**b**) Flowchart of LabVIEW program.

The microcontroller uses Arduino integrated development environment and uses C/C++ language to write programs. The whole program adopts modular design, mainly including initialization program, main program, ultrasonic sensor data acquisition program, and serial interrupt service program. Figure 6a shows the flowchart of Arduino.

#### 3.3.2. LabVIEW Program Design

The system software of the upper computer in the experiment is developed based on the LabVIEW platform and consists of two parts: the program interface of the front panel and the block diagram of the back panel. The flowchart is depicted as shown in Figure 6b. The LabVIEW program collects data through serial communication, and the background software implements data analysis and processing. Finally, the detection results and corresponding measurement curves are output through the front panel interface window.

**Figure 7:**
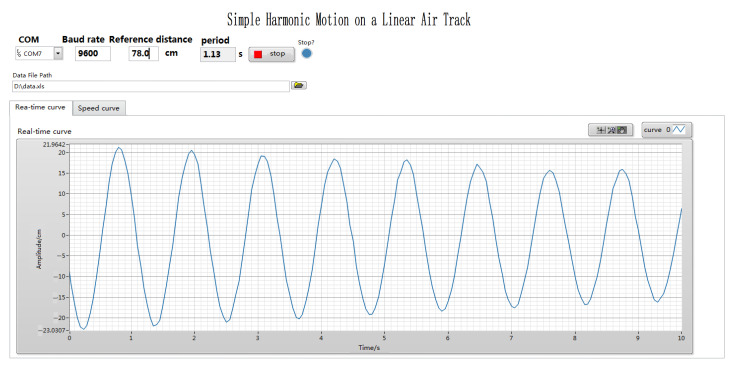
The amplitude time curve displayed on the front panel of LabVIEW.

**Figure 8:**
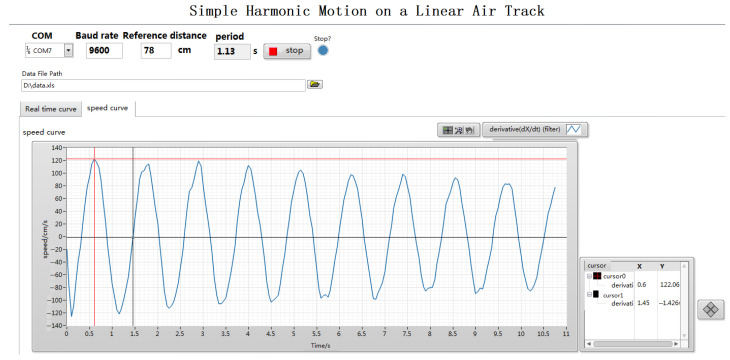
The speed time curve displayed on the front panel of LabVIEW.

**Figure 9:**
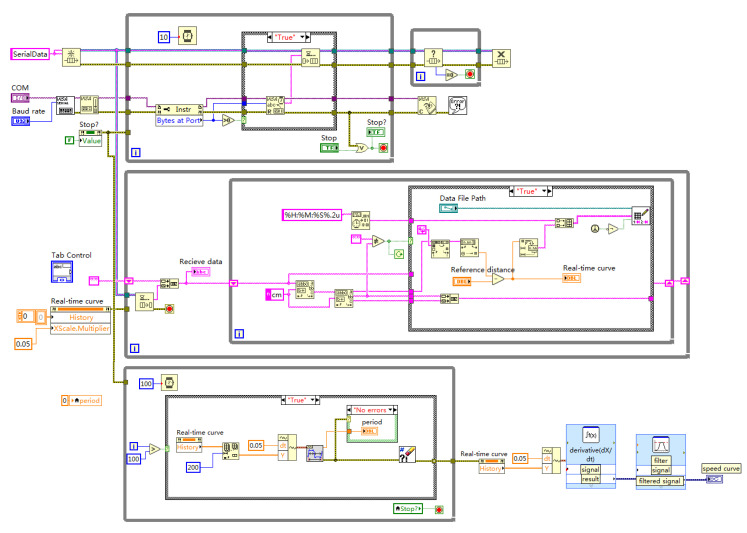
The program on the back board of LabVIEW.

The front panels of LabVIEW, namely the program interface, are shown in Figure 7 and Figure 8, including the setting of experimental parameters and the display of measurement results. Figure 7 shows the amplitude time curve collected by the ultrasonic sensor, while Figure 8 shows the velocity time curve, which is obtained by differentiation. As shown in Figure 9, the program design of the back panel of LabVIEW corresponds to the functions of the front panel one by one, which is mainly composed of the serial port configuration module, data display export module, and data processing module. This section uses the NI-VISA window configuration assistant to connect the LabVIEW program in the lower computer to the computer. Before this, the parameters of the serial port are set, including the baud rate, serial port name, data sending bits, and data flag bits. The data read is stored in the LabVIEW program and can be exported in text or excel format.

## 4. Experimental Results

**Figure 10:**
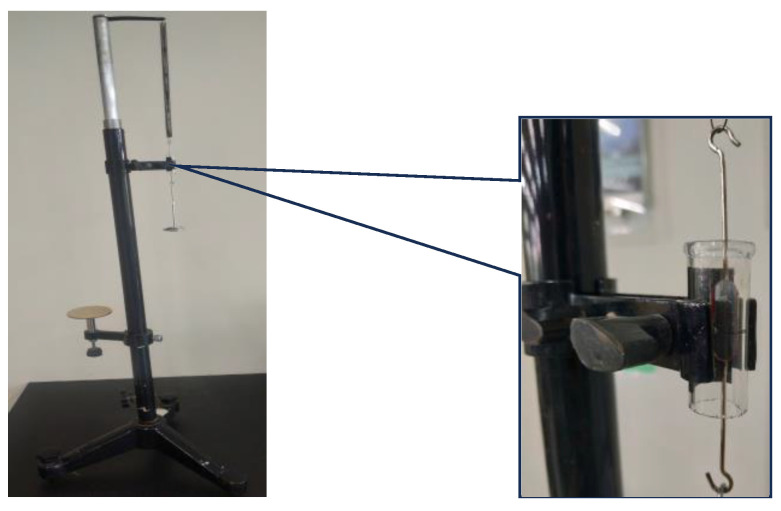
The stiffness coefficient *k* of the spring is measured using the Jolly scale.

**Figure 11:**
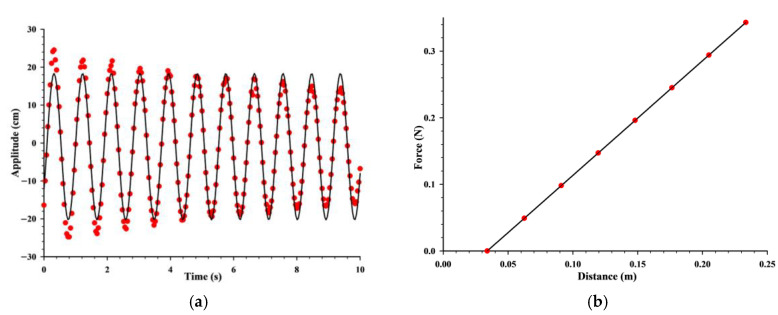
The data curve fitting represented using Sigmaplot 15 (the red dots denote the original data points, while the black line represents the fitted curve). (**a**) The relationship between amplitude and time. (**b**) The variation of force with distance.

As shown in Figure 10, two linear springs with similarity stiffness factors are measured with the Jolly scale. Adjust the Jolly scale knob so that the horizontal line on the lower end of the spring, the horizontal line on the glass tube, and the image of the horizontal line in the small mirror can be coincided—this is abbreviated as the “three line overlap”. Record the corresponding scale reading *l*_0_. Then, add 5 g of weight to the weighing plate, align the three lines, and record the reading *l*_1_. Continue to add 5 g of weight and record the reading *l_2_* and so on. Record the reading *l*_7_ until the 35 g weight is added. Measure the corresponding reading of spring 2 using the same method and record the reading in Table 1. Among them, $\bar{\delta l_{1}}=\frac{\left(l_{7}-l_{3}\right)+\left(l_{6}-l_{2}\right)+\left(l_{5}-l_{1}\right)+\left(l_{4}-l_{0}\right)}{4}=113.95$ mm is the average difference of the spring’s elongation. ${\bar{k}}_{1}=\frac{\Delta mg}{\bar{\delta l_{1}}}=\frac{4\times 5\times 9.8}{113.95}=1.72\;$ N/m is the average calculated stiffness coefficient of the spring constant. The measured and processed results are listed in Table 1. The raw data of the distance from the ultrasonic sensor to the slider as a time function are imported into Sigmaplot 15 for data analysis. The Sigmaplot 15 is a scientific plotting software primarily used for data visualization, statistical analysis, and scientific graphic presentation. As shown in Figure 11a, The curve fitting adopts a nonlinear model. A model is defined in the form of $y_{0}+A\sin\left(\frac{2\pi t}{T}-\varphi \right)$. The fitting curve equation is $y=-0.3236+19.321\sin\left(\frac{2\pi }{1.131}x-0.624\right)$. In this situation, the initial phase amplitude (φ) is 0.624 ± 0.011 rad, the amplitude (*A*) is 0.19321 ± 0.00211 m, and the period (*T*) is 1.131 ± 0.041 s. The coefficient of determination (*R*^2^) is calculated to be 0.9818, indicating a strong correlation between the sinusoidal model used for curve fitting and the experimental data.

The linear regression analysis equation shown in Figure 11b is F=1.7x−0.058, providing a slope value of 1.7 N/m and representing the spring constant (*k*). The measured mass of the slider is 110.0 g. Indirect measurement of vibration period (*T*) can be calculated using Equation (6). $T=2\pi \sqrt{\frac{m}{k_{1}+k_{2}}}=2\pi \sqrt{\frac{110\times 10^{-3}}{1.72+1.72}}=1.123\;$ s. In Figure 11a, the period (*T*) value obtained from fitting the data curve is 1.131 ± 0.041 s. So, the experimental results indicate that the measured value of the period (*T*) is consistent with the theoretical calculation.

Analyzing Figure 7, it can be concluded that within nearly five cycles, the maximum amplitude of the system is essentially equal, and at the maximum amplitude, the velocity of the slider is zero. Therefore, the sum of the kinetic energy and potential energy of the system is equal to the magnitude of the potential energy, and the mechanical energy of the system tends to be equal. In order to better verify the mechanical energy conservation of the system, LabVIEW was used to analyze the velocity time image of the slider movement, as shown in Figure 8. On the diagram, the speed at any time can be read through the cross-cursor positioning. From Figure 8, it can be seen that $v_{\max}=1.2\;$ m/s and m=0.11  kg, so the maximum kinetic energy $E_{k}=mv_{\max}^{2}/2=0.11\times 1.2^{2}÷2=0.0792$ J. From Figure 7, it can be seen that the amplitude A=0.215 m and $k_{1}=k_{2}=1.72$ N/m, so the maximum potential energy $E_{p}=\frac{1}{2}\left(k_{1}+k_{2}\right)A^{2}=\frac{1}{2}\times \left(1.72+1.72\right)\times 0.215^{2}=0.0795$ J. From Figure 8, it can be seen that the speed at the position of half the amplitude is $v_{A/2}=1.03$ m/s, so the total energy at the position of half the amplitude $E=E_{k}^{\prime }+E_{p}^{\prime }=mv^{2}/2+kx^{2}/2=0.0791$ J. From the above calculation, it can be concluded that $E\approx E_{k}\approx E_{p}$. The above results verify the conservation of mechanical energy in harmonic vibration.

The harmonic vibration experiment after digital transformation can not only perform point measurement, but also support continuous measurement, achieving real-time measurement of dynamic amplitude, data visualization, and a more convenient observation of experimental details and quantitative calculation. From the amplitude time curve in Figure 7, it can be clearly observed that due to air resistance and other reasons, the amplitude slowly decreases over time, indicating a damping vibration. The amplitude curve is a sine wave, and the vibration period is independent of the amplitude, which is consistent with physical theory.

## 5. Conclusions

Based on the LabVIEW interface of Arduino, a digital harmonic vibration experimental device has been developed that is modern, cost-effective, simple, and innovative. The amplitude time curve measured by the HC-SR04 ultrasonic sensor allows students to visually observe harmonic vibrations, improving the visualization and automation of the experiment. The trajectory of the slider movement on the air track can be accurately recorded by LabVIEW, and its motion data can be directly sampled with a large amount of data, making it convenient for selection and processing. The results verify the mechanical energy conservation of a spring oscillator system in a short time. The experimental data are in good agreement with the theoretical model. During the development process of this experimental setup, teachers and students invisibly promoted the achievement of information literacy and core competencies in physics. The experimental system based on LabVIEW interface for Arduino has strong portability and can develop multiple digital experiments on the same experimental equipment, making it highly valuable for promotion. 

## Figures and Tables

**Table 1:** The measurement results of the two springs’ stiffness coefficient.

No.	m (g)	Spring 1 Length (mm)	Spring 1 Elongation (mm)	Spring 1 Constant k (N/m)	Spring 2 Length (mm)	Spring 2 Elongation (mm)	Spring 2 Constant k (N/m)
0	0	$l_{0}$ = 33.9	$\delta l_{1}$ = $l_{4}$−$l_{0}$ = 114.1	$\bar{k_{1}}=1.72$	${l^{\prime }}_{0}$ = 34.8	$\delta {l^{\prime }}_{1}$ = ${l^{\prime }}_{4}$−${l^{\prime }}_{0}$ = 114.2	$\bar{k_{2}}=1.72$
1	5	$l_{1}$ = 62.5	$\delta l_{2}$ = $l_{5}$−$l_{1}$ = 113.9		${l^{\prime }}_{1}$ = 63.4	$\delta {l^{\prime }}_{2}$ = ${l^{\prime }}_{5}$−${l^{\prime }}_{1}$ = 114.0	
2	10	$l_{2}$ = 91.0	$\delta l_{3}$ = $l_{6}$−$l_{2}$ = 113.9		${l^{\prime }}_{2}$ = 92.0	$\delta {l^{\prime }}_{3}$ = ${l^{\prime }}_{6}$−${l^{\prime }}_{2}$ = 113.8	
3	15	$l_{3}$ = 119.5	$\delta l_{4}$ = $l_{7}$−$l_{3}$ = 113.9		${l^{\prime }}_{3}$ = 120.5	$\delta {l^{\prime }}_{4}$ = ${l^{\prime }}_{7}$−${l^{\prime }}_{3}$ = 113.8	
4	20	$l_{4}$ = 148.0	$\bar{\delta l_{1}}$ = 113.95		${l^{\prime }}_{4}$ = 149.0	$\bar{\delta l_{2}}$ = 113.95	
5	25	$l_{5}$ = 176.4			${l^{\prime }}_{5}$ = 177.4		
6	30	$l_{6}$ = 204.9			${l^{\prime }}_{6}$ = 205.8		
7	35	$l_{7}$ = 233.4			${l^{\prime }}_{7}$ = 234.3		

## Data Availability

Data are contained within the article.
